# Diaqua­bis[3-(2-sulfanylphen­yl)prop-2-enoato]zinc(II) dihydrate

**DOI:** 10.1107/S1600536809034473

**Published:** 2009-09-09

**Authors:** Qiang Wang, Jian Hou, Li-Jun Wang, Qing-Fu Zeng

**Affiliations:** a Engineering Research Center for Clean Production of Textile Dyeing and Printing, Ministry of Education, Wuhan 430073, People’s Republic of China

## Abstract

In the title compound, [Zn(C_9_H_7_O_2_S)_2_(H_2_O)_2_]·2H_2_O, the Zn^II^ atom (site symmetry 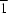) is four-coordinated by two O atoms from 3-(2-sulfanylphen­yl)prop-2-enoate anions and two aqua O atoms in a slightly distorted ZnO_4_ square-planar arrangement. In the crystal, O—H⋯O hydrogen bonds help to establish the packing.

## Related literature

For background to coordination networks, see: Cheng *et al.*, (2006 ▶). For reference structural data, see: Allen *et al.* (1987 ▶).
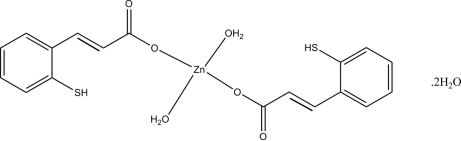


## Experimental

### 

#### Crystal data

[Zn(C_9_H_7_O_2_S)_2_(H_2_O)_2_]·2H_2_O
*M* *_r_* = 495.85
Monoclinic, 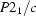

*a* = 18.4398 (5) Å
*b* = 7.7188 (3) Å
*c* = 7.3258 (2) Å
β = 98.578 (2)°
*V* = 1031.04 (6) Å^3^
*Z* = 2
Mo *K*α radiation
μ = 1.44 mm^−1^
*T* = 298 K
0.30 × 0.20 × 0.14 mm

#### Data collection

Enraf–Nonius CAD-4 diffractometer
Absorption correction: ψ scan (North *et al.*, 1968 ▶) *T*_min_ = 0.673, *T*_max_ = 0.824
6272 measured reflections
1811 independent reflections
1441 reflections with *I* > 2σ(*I*)
*R*_int_ = 0.092
200 standard reflections every 3 reflections intensity decay: 1%

#### Refinement

*R*[*F* ^2^ > 2σ(*F* ^2^)] = 0.067
*wR*(*F* ^2^) = 0.185
*S* = 1.06
1811 reflections
146 parameters
6 restraints
H atoms treated by a mixture of independent and constrained refinement
Δρ_max_ = 0.93 e Å^−3^
Δρ_min_ = −0.88 e Å^−3^

### 

Data collection: *CAD-4 Software* (Enraf–Nonius, 1989 ▶); cell refinement: *CAD-4 Software*; data reduction: *XCAD4* (Harms & Wocadlo, 1995 ▶); program(s) used to solve structure: *SHELXS97* (Sheldrick, 2008 ▶); program(s) used to refine structure: *SHELXL97* (Sheldrick, 2008 ▶); molecular graphics: *SHELXTL* (Sheldrick, 2008 ▶); software used to prepare material for publication: *SHELXTL*.

## Supplementary Material

Crystal structure: contains datablocks global, I. DOI: 10.1107/S1600536809034473/hb5073sup1.cif

Structure factors: contains datablocks I. DOI: 10.1107/S1600536809034473/hb5073Isup2.hkl

Additional supplementary materials:  crystallographic information; 3D view; checkCIF report

## Figures and Tables

**Table 1:** Selected bond lengths (Å)

Zn1—O1	1.969 (4)
Zn1—O3	1.953 (4)

**Table 2:** Hydrogen-bond geometry (Å, °)

*D*—H⋯*A*	*D*—H	H⋯*A*	*D*⋯*A*	*D*—H⋯*A*
O3—H3*A*⋯O4^i^	0.82 (4)	2.56 (5)	3.033 (7)	118 (5)
O3—H3*B*⋯O1^ii^	0.82 (4)	2.44 (5)	3.221 (6)	159 (4)
O4—H4*A*⋯O2	0.83 (3)	1.95 (4)	2.716 (7)	155 (5)
O4—H4*B*⋯O2^iii^	0.83 (4)	2.30 (6)	2.951 (7)	136 (4)

Symmetry codes: (i) 
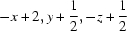
; (ii) 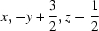; (iii) 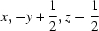.

**Fig. 1.**
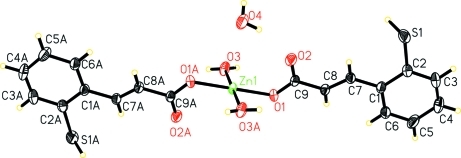
The molecular structure of (I) showing 30% probability displacement ellipsoids. Atoms with the suffix A are generated by the symmetry operation (2–x, 1–y, 1–z).

## supplementary crystallographic information

### Comment

There has been much research interest in acid metal complexes due to their molecular architectures and biological activities (e.g. Cheng *et al.*, 2006). In this work, we report here the crystal structure of the title compound, (I). In (I), all bond lengths are within normal ranges (Allen *et al.*, 1987) (Fig. 1). The Zn^II^ atom is four-coordinated by two O atoms from the 3-(2-sulfanylphenyl)propanoate and two O atoms from the water molecules, forming a slightly distorted square-planar coordination.

### Experimental

A mixture of 3-(2-sulfanylphenyl)propanoic acid (364 mg, 2 mmol) and ZnCl_2_ (1 mmol, 134 mg) in methanol (10 ml) was stirred for 3 h. After keeping the filtrate in air for 7 d, colourless blocks of (I) were formed.

### Refinement

The water H atoms were located in a difference map and their positions were refined with restraints of O—H = 0.82 (1)Å. The other H atoms were positioned geometrically (C—H = 0.93Å, S—H = 1.20 Å) and refined as riding, with *U*_iso_(H) = 1.2*U*_eq_(carrier).

### Crystal data

**Table:**

[Zn(C_9_H_7_O_2_S)_2_(H_2_O)_2_]·2H_2_O	*F*(000) = 512
*M**_r_* = 495.85	*D*_x_ = 1.597 Mg m^−^^3^
Monoclinic, *P*2_1_/*c*	Mo *K*α radiation, λ = 0.71073 Å
Hall symbol: -P 2ybc	Cell parameters from 25 reflections
*a* = 18.4398 (5) Å	θ = 9–12°
*b* = 7.7188 (3) Å	µ = 1.44 mm^−^^1^
*c* = 7.3258 (2) Å	*T* = 298 K
β = 98.578 (2)°	Block, colourless
*V* = 1031.04 (6) Å^3^	0.30 × 0.20 × 0.14 mm
*Z* = 2	

### Data collection

**Table:**

Enraf–Nonius CAD-4 diffractometer	1441 reflections with *I* > 2σ(*I*)
Radiation source: fine-focus sealed tube	*R*_int_ = 0.092
graphite	θ_max_ = 25.0°, θ_min_ = 2.2°
ω/2θ scans	*h* = −21→18
Absorption correction: ψ scan (North *et al.*, 1968)	*k* = −9→8
*T*_min_ = 0.673, *T*_max_ = 0.824	*l* = −8→8
6272 measured reflections	200 standard reflections every 3 reflections
1811 independent reflections	intensity decay: 1%

### Refinement

**Table:**

Refinement on *F*^2^	Primary atom site location: structure-invariant direct methods
Least-squares matrix: full	Secondary atom site location: difference Fourier map
*R*[*F*^2^ > 2σ(*F*^2^)] = 0.067	Hydrogen site location: inferred from neighbouring sites
*wR*(*F*^2^) = 0.185	H atoms treated by a mixture of independent and constrained refinement
*S* = 1.06	*w* = 1/[σ^2^(*F*_o_^2^) + (0.1071*P*)^2^ + 0.9875*P*] where *P* = (*F*_o_^2^ + 2*F*_c_^2^)/3
1811 reflections	(Δ/σ)_max_ = 0.001
146 parameters	Δρ_max_ = 0.93 e Å^−^^3^
6 restraints	Δρ_min_ = −0.88 e Å^−^^3^

### Special details

**Table:**

Geometry. All e.s.d.'s (except the e.s.d. in the dihedral angle between two l.s. planes) are estimated using the full covariance matrix. The cell e.s.d.'s are taken into account individually in the estimation of e.s.d.'s in distances, angles and torsion angles; correlations between e.s.d.'s in cell parameters are only used when they are defined by crystal symmetry. An approximate (isotropic) treatment of cell e.s.d.'s is used for estimating e.s.d.'s involving l.s. planes.
Refinement. Refinement of *F*^2^ against ALL reflections. The weighted *R*-factor *wR* and goodness of fit *S* are based on *F*^2^, conventional *R*-factors *R* are based on *F*, with *F* set to zero for negative *F*^2^. The threshold expression of *F*^2^ > σ(*F*^2^) is used only for calculating *R*-factors(gt) *etc*. and is not relevant to the choice of reflections for refinement. *R*-factors based on *F*^2^ are statistically about twice as large as those based on *F*, and *R*- factors based on ALL data will be even larger.

### Fractional atomic coordinates and isotropic or equivalent isotropic displacement parameters (Å^2^)

**Table:**

	*x*	*y*	*z*	*U*_iso_*/*U*_eq_	
C1	0.6975 (3)	0.9228 (8)	0.6126 (7)	0.0401 (13)	
C2	0.6254 (3)	0.8922 (8)	0.6435 (8)	0.0442 (14)	
C3	0.5803 (4)	1.0275 (10)	0.6681 (10)	0.0590 (19)	
H3	0.5327	1.0042	0.6892	0.071*	
C4	0.6020 (4)	1.1926 (10)	0.6630 (11)	0.070 (2)	
H4	0.5700	1.2818	0.6811	0.084*	
C5	0.6739 (4)	1.2307 (10)	0.6300 (10)	0.066 (2)	
H5	0.6898	1.3446	0.6253	0.079*	
C6	0.7196 (3)	1.0945 (8)	0.6051 (9)	0.0500 (16)	
H6	0.7669	1.1183	0.5825	0.060*	
C7	0.7479 (3)	0.7796 (7)	0.5892 (7)	0.0374 (12)	
H7	0.7326	0.6685	0.6150	0.045*	
C8	0.8132 (3)	0.7942 (7)	0.5345 (7)	0.0361 (12)	
H8	0.8281	0.9030	0.5003	0.043*	
C9	0.8630 (3)	0.6463 (7)	0.5254 (7)	0.0354 (12)	
H3A	1.0261 (19)	0.464 (7)	0.180 (6)	0.043*	
H4A	0.866 (2)	0.294 (4)	0.419 (8)	0.043*	
H3B	0.964 (2)	0.558 (6)	0.165 (6)	0.043*	
H4B	0.861 (2)	0.133 (5)	0.346 (8)	0.043*	
O1	0.92576 (19)	0.6850 (5)	0.4821 (5)	0.0401 (9)	
O2	0.8441 (3)	0.4964 (5)	0.5594 (7)	0.0530 (12)	
O3	0.9908 (3)	0.4908 (6)	0.2311 (6)	0.0499 (11)	
O4	0.8892 (3)	0.2064 (6)	0.3989 (8)	0.0666 (13)	
S1	0.59232 (9)	0.6819 (2)	0.6468 (3)	0.0601 (6)	
H1	0.5970	0.6126	0.5024	0.090*	
Zn1	1.0000	0.5000	0.5000	0.0327 (4)	

### Atomic displacement parameters (Å^2^)

**Table:**

	*U*^11^	*U*^22^	*U*^33^	*U*^12^	*U*^13^	*U*^23^
C1	0.038 (3)	0.044 (3)	0.039 (3)	0.008 (3)	0.008 (2)	−0.006 (3)
C2	0.040 (3)	0.050 (4)	0.045 (3)	0.005 (3)	0.013 (3)	0.001 (3)
C3	0.040 (4)	0.071 (5)	0.069 (5)	0.018 (3)	0.018 (3)	0.003 (4)
C4	0.063 (5)	0.062 (5)	0.088 (5)	0.033 (4)	0.020 (4)	−0.004 (4)
C5	0.078 (5)	0.042 (4)	0.079 (5)	0.013 (4)	0.021 (4)	−0.012 (3)
C6	0.040 (3)	0.041 (4)	0.072 (4)	0.005 (3)	0.017 (3)	−0.004 (3)
C7	0.034 (3)	0.034 (3)	0.045 (3)	0.005 (2)	0.008 (2)	0.002 (2)
C8	0.037 (3)	0.032 (3)	0.040 (3)	0.005 (2)	0.011 (2)	0.003 (2)
C9	0.037 (3)	0.038 (3)	0.032 (3)	0.007 (2)	0.009 (2)	−0.002 (2)
O1	0.032 (2)	0.045 (2)	0.045 (2)	0.0091 (16)	0.0099 (17)	0.0037 (17)
O2	0.052 (3)	0.035 (3)	0.075 (3)	0.0035 (19)	0.019 (2)	0.000 (2)
O3	0.062 (3)	0.052 (3)	0.040 (2)	0.017 (2)	0.021 (2)	0.0060 (18)
O4	0.074 (3)	0.049 (3)	0.082 (4)	0.014 (2)	0.030 (3)	−0.004 (3)
S1	0.0465 (9)	0.0555 (11)	0.0842 (13)	−0.0072 (8)	0.0287 (9)	0.0035 (9)
Zn1	0.0348 (6)	0.0360 (6)	0.0300 (5)	0.0075 (4)	0.0139 (4)	0.0046 (3)

### Geometric parameters (Å, °)

**Table:**

C1—C6	1.390 (10)	C8—C9	1.473 (7)
C1—C2	1.402 (8)	C8—H8	0.9300
C1—C7	1.470 (8)	C9—O2	1.245 (6)
C2—C3	1.364 (9)	C9—O1	1.280 (6)
C2—S1	1.735 (7)	Zn1—O1	1.969 (4)
C3—C4	1.338 (10)	Zn1—O3	1.953 (4)
C3—H3	0.9300	O3—H3A	0.824 (10)
C4—C5	1.413 (11)	O3—H3B	0.823 (10)
C4—H4	0.9300	O4—H4A	0.821 (10)
C5—C6	1.376 (9)	O4—H4B	0.819 (10)
C5—H5	0.9300	S1—H1	1.2000
C6—H6	0.9300	Zn1—O3^i^	1.953 (4)
C7—C8	1.330 (7)	Zn1—O1^i^	1.969 (4)
C7—H7	0.9300		
			
C6—C1—C2	117.2 (5)	C1—C7—H7	117.0
C6—C1—C7	121.3 (5)	C7—C8—C9	123.2 (5)
C2—C1—C7	121.5 (6)	C7—C8—H8	118.4
C3—C2—C1	120.3 (6)	C9—C8—H8	118.4
C3—C2—S1	119.4 (5)	O2—C9—O1	123.9 (5)
C1—C2—S1	120.3 (5)	O2—C9—C8	121.0 (5)
C4—C3—C2	122.3 (7)	O1—C9—C8	115.0 (5)
C4—C3—H3	118.9	C9—O1—Zn1	117.3 (4)
C2—C3—H3	118.9	Zn1—O3—H3A	121 (4)
C3—C4—C5	119.7 (6)	Zn1—O3—H3B	121 (4)
C3—C4—H4	120.1	H3A—O3—H3B	110.2 (18)
C5—C4—H4	120.1	H4A—O4—H4B	110.3 (18)
C6—C5—C4	118.2 (7)	C2—S1—H1	109.5
C6—C5—H5	120.9	O3^i^—Zn1—O3	180.0
C4—C5—H5	120.9	O3^i^—Zn1—O1^i^	90.25 (17)
C5—C6—C1	122.3 (6)	O3—Zn1—O1^i^	89.75 (17)
C5—C6—H6	118.8	O3^i^—Zn1—O1	89.75 (17)
C1—C6—H6	118.8	O3—Zn1—O1	90.25 (17)
C8—C7—C1	126.0 (5)	O1^i^—Zn1—O1	180.0
C8—C7—H7	117.0		
			
C6—C1—C2—C3	−1.1 (9)	C7—C1—C6—C5	−178.8 (6)
C7—C1—C2—C3	178.8 (6)	C6—C1—C7—C8	−10.0 (9)
C6—C1—C2—S1	177.7 (4)	C2—C1—C7—C8	170.1 (6)
C7—C1—C2—S1	−2.4 (8)	C1—C7—C8—C9	175.9 (5)
C1—C2—C3—C4	0.4 (11)	C7—C8—C9—O2	4.1 (8)
S1—C2—C3—C4	−178.5 (6)	C7—C8—C9—O1	−175.4 (5)
C2—C3—C4—C5	0.4 (12)	O2—C9—O1—Zn1	−7.6 (7)
C3—C4—C5—C6	−0.4 (11)	C8—C9—O1—Zn1	171.9 (3)
C4—C5—C6—C1	−0.4 (10)	C9—O1—Zn1—O3^i^	−70.7 (4)
C2—C1—C6—C5	1.2 (9)	C9—O1—Zn1—O3	109.3 (4)

Symmetry codes: (i) −*x*+2, −*y*+1, −*z*+1.

### Hydrogen-bond geometry (Å, °)

**Table:**

*D*—H···*A*	*D*—H	H···*A*	*D*···*A*	*D*—H···*A*
O3—H3A···O4^ii^	0.82 (4)	2.56 (5)	3.033 (7)	118 (5)
O3—H3B···O1^iii^	0.82 (4)	2.44 (5)	3.221 (6)	159 (4)
O4—H4A···O2	0.83 (3)	1.95 (4)	2.716 (7)	155 (5)
O4—H4B···O2^iv^	0.83 (4)	2.30 (6)	2.951 (7)	136 (4)

Symmetry codes: (ii) −*x*+2, *y*+1/2, −*z*+1/2; (iii) *x*, −*y*+3/2, *z*−1/2; (iv) *x*, −*y*+1/2, *z*−1/2.
